# Nanostructured Carbonated Hydroxyapatite Associated to rhBMP-2 Improves Bone Repair in Rat Calvaria

**DOI:** 10.3390/jfb11040087

**Published:** 2020-12-04

**Authors:** Thiago Schneider Werner Vianna, Suelen Cristina Sartoretto, Adriana Terezinha Neves Novellino Alves, Rodrigo Figueiredo de Brito Resende, Carlos Fernando de Almeida Barros Mourão, Jose de Albuquerque Calasans-Maia, Victor R. Martinez-Zelaya, Alexandre Malta Rossi, Jose Mauro Granjeiro, Monica Diuana Calasans-Maia, Rafael Seabra Louro

**Affiliations:** 1 Pos-Graduation Program, Dentistry School, Universidade Federal Fluminense, Rio de Janeiro 24020-140, Brazil; thiagocirurgia@gmail.com (T.S.W.V.); mouraocf@gmail.com (C.F.d.A.B.M.); jmgranjeiro@gmail.com (J.M.G.); 2 Oral Surgery Department, Universidade Veiga de Almeida, Rio de Janeiro 20271-020, Brazil; susartoretto@hotmail.com; 3 Oral Surgery Department, Universidade Iguaçu, Rio de Janeiro 26260-045, Brazil; rodrigoodonto21@hotmail.com; 4 Oral Diagnosis Department, Dentistry School, Universidade Federal Fluminense, Rio de Janeiro 24020-140, Brazil; aterezinhanovellino@gmail.com; 5 Oral Surgery Department, Dentistry School, Universidade Federal Fluminense, Rio de Janeiro 24020-140, Brazil; dr.rafaelseabra@gmail.com; 6 Orthodontic Department, Dentistry School, Universidade Federal Fluminense, Rio de Janeiro 24020-140, Brazil; josecalasans@id.uff.br; 7 Brazilian Synchrotron Light Laboratory (LNLS), Brazilian Center for Research in Energy and Materials (CNPEM), São Paulo 13083-970, Brazil; victor.martinez@lnls.br; 8 Department of Condensed Matter, Applied Physics and Nanoscience, Brazilian Center for Research in Physics, Rio de Janeiro 22290-180, Brazil; rossi@cbpf.br; 9 Directory of Life Sciences Applied Metrology, Instituto Nacional de Metrologia, Qualidade e Tecnologia (INMETRO), Duque de Caxias 25250-020, Brazil

**Keywords:** nanostructured carbonated hydroxyapatite, rhBMP-2, critical-size defect, bone regeneration

## Abstract

Many biomaterials are used for Bone Morphogenetic Proteins (BMPs) delivery in bone tissue engineering. The BMP carrier system’s primary function is to hold these growth factors at the wound’s site for a prolonged time and provide initial support for cells to attach and elaborate the extracellular matrix for bone regeneration. This study aimed to evaluate the nanostructured carbonated hydroxyapatite microspheres (nCHA) as an rhBMP-2 carrier on rats calvaria. A total of fifteen male Wistar rats were randomly divided into three groups (*n* = 5): clot (control group), rhBMP-2 associated with collagen membrane (COL/rhBMP-2) or associated with the microspheres (nCHA/rhBMP-2). After 45 days, the calvaria defect samples were evaluated through histological, histomorphometric, and SR-µCT analyses to investigate new-formed bone and connective tissue volume densities. The descriptive histological analysis showed that nCHA/rhBMP-2 improved bone formation compared to other groups. These results were confirmed by histomorphometric and SR-µCT analysis that showed substantially defect area filling with a higher percentage of newly formed (36.24 ± 6.68) bone than those with the COL/rhBMP-2 (0.42 ± 0.40) and Clot (3.84 ± 4.57) (*p* < 0.05). The results showed that nCHA is an effective carrier for rhBMP-2 encouraging bone healing and an efficient alternative to collagen membrane for rhBMP-2 delivery.

## 1. Introduction

The use of biomaterials as a bone substitute represents an alternative to autogenous grafts and is permanently the target of scientific research; for this reason, many of the recent studies seek to improve the material properties to become more similar and an alternative to autografts. The autogenous graft is still considered the gold standard in reconstructing bone critical-size defects since it presents osteoinductive and osteogenic properties. However, its use depends on another surgical site as a donor area, and there is an unpredictability quantity of the graft due to resorption during the healing period [1].

Synthetic biomaterials represent a long-awaited hope in bone reconstruction surgery [1,2]. A series of research is being developed to increase its security, affectivity, and similarity to natural bone. Hydroxyapatite (Ca_10_(PO_4_)_6_(OH)_2_), (HA), the main constituent of the inorganic bone and teeth composition, is widely researched as bone substitute biomaterial [2,3]. Meanwhile, the sintered HA’s crystallinity impacts its nanostructural characteristics, which decreases its bioabsorption ability [4]. Previous studies proposed the chemical modification of HA replacing the phosphate group (PO_4_^2−^) with the carbonate group (CO_3_^2−^) thus synthesizing the nanostructured carbonated hydroxyapatite (nCHA) [5,6,7,8,9]. Besides that, the absence of thermic treatment during the synthesis process ensures its nanometric characteristics promote exciting changes in the material properties, mainly compromising their solubility after implantation, thermal stability, particle size, and morphology in physiology systems [6,7,8,9].

The nCHA has shown favorable results in previous preclinical and clinical studies in its original form [7] or associated with metals [8,9,10] with growth factors [11] or with antibiotics [12]. Its microspheres morphology, formed from a reaction with sodium alginate, allows easy manipulation, the formation of internal pores [12], and less inflammatory response than needle shaped HAs [13]. The literature shows that the needle morphology of biomaterial prolonged inflammatory response is compared to spherical-shaped nanoparticles and suggests that these might be detrimental in promoting successful tissue remodeling [13]. Additionally, the ability of the controlled delivery of therapeutic substances by nCHA has been investigated and proposed as a promising approach to prevent and control local inflammatory processes and chronic infections [12].

The rhBMP-2 is a cytokine that plays an essential role in skeletal development [14]. It can induce differentiation of mesenchymal stem cells and stem cells into osteogenic cells, producing bone [15,16,17].

A wide range of biomaterials have been studied as BMPs carriers for bone tissue regeneration, as natural and synthetic polymers, titanium, and ceramics composites [17]. A BMP carrier should preferably induce a minimal inflammatory response, be completely biodegradable with adequate porosity for infiltration and proliferation of cells at the new bone tissue site [18]; should prevent the BMP degradation maintaining its bioactivity and allow a sustained release in a controlled way to promote the formation of new bone at the defect’s site [19,20,21]. It should be easily sterilized, easy to handle, stable when stored, and be commercially viable, enabling an upscale production [17].

The association of rhBMP-2 with a biomaterial may reduce the morbidity of the surgical procedure related to autologous graft, avoiding the postoperative discomfort and the probability of complications.

Considering that the nCHA microspheres have a high surface area and a known porosity [12], we hypothesized that the morphology characteristics of the nCHA microspheres might provide an excellent carrier, associating the osteoconduction and scaffold properties of calcium phosphate materials with the osteoinduction activity of recombinant human bone morphogenetic protein 2.

Based on this context, this study aimed to evaluate the suitability of the nCHA to carry rhBMP-2 on bone regeneration in contrast to collagen-associated rhBMP-2. We chose critical-size rats calvaria defects to assess the newly formed bone through histology, histomorphometry, and Synchrotron Radiation-based X-ray Microtomography (SR-µCT) analyses.

## 2. Materials and Methods

### 2.1. Ethical Aspects and Study Design

The Ethics Committee on Animal Use of Universidade Federal Fluminense (CEUA/UFF-834) approved this study. The experiments were conducted according to guidelines of the NIH Guide for the Care and Use of Laboratory Animals following the Brazilian Directive for the Care and Use of Animals for Scientific and Didactic Purposes—DBCA and the CONCEA Euthanasia Practice Guidelines. According to ARRIVE guidelines [22], this experiment was described and supplemented by PREPARE [23] regarding relevant items.

### 2.2. Sample Size Calculation

A significant effect of 15% or more 45 days after surgery in the new-formed bone (primary outcome) would be of interest. Considering previous rates [24] for new-formed bone at 45 days post-surgery in the control group and intervention group of 50% and 41%, respectively, with a two-sided significance of 0.05 and a power of 0.9, according the site https://www.sealedenvelope.com/power/continuous-superiority/ [Accessed on 10 October 2020], this study required a total of five animals [25].

### 2.3. Animal Model and Groups

A total of fifteen adult male rats (*Rattus norvegicus albinus*, Wistar, Philadelphia, PA, USA), weighing between 300 and 350 g at six months old, were used. The animals were provided by the Laboratory Animal Center (NAL), located at the Fluminense Federal University, Niterói, Rio de Janeiro, Brazil. The animals were kept in isolators with a maximum of 2 animals in each and were fed with pelleted feed and free water. The ambient temperature was maintained between 16 and 20 °C, as it was ideal for rats’ growth. A photo-period control of 12 h light and 12 h dark was established, providing the correct metabolic cycle. A senior veterinarian monitored the nutritional parameters, animal care, and pre- and postoperative fasting of the animals.

The animals were randomly divided (*n* = 5) into three groups according to treatment: clot, representing the animal that underwent sham surgery only to simulate surgical stress (Control group; CLOT); collagen membrane and Human Recombinant Morphogenetic Protein (rhBMP-2), both available from the Infuse kit (Infuse^®^ Bone Graft, Medtronic, Memphis, TN, USA) (COL/rhBMP-2 group) and nanostructured carbonated hydroxyapatite microspheres (nCHA) with rhBMP-2 (Infuse^®^ Bone Graft, Medtronic, Memphis, TN, USA) (nCHA/rhBMP-2 group). The experimental period was 45 days.

### 2.4. Nanostructured Carbonated Hydroxyapatite (nCHA) Synthesis

Microspheres of nCHA (425 to 600 µm) containing sodium alginate were synthesized using a wet precipitation method containing 6% (by weight) CO_3_^2−^, with stoichiometry (1.6 < Ca/P < 2.0) according to previously published protocol [8,11]. The nanostructured powders of carbonated hydroxyapatite were precipitated through the addition of an aqueous (NH_4_)_2_HPO_4_ solution to a solution containing Ca(NO_3_)_2_, Ca(CO_3_)_2_·4H_2_O, and (NH_4_)_2_CO_3_ at a pH of 9.0, followed by stirring of the suspension for 3 h at 37 °C. The precipitate was then separated by filtration, repeatedly washed with boiling deionized water, and subsequently dried at 100 °C for 24 h. The dried powder was dispersed in a 10 mg/mL aqueous sodium alginate solution to achieve an alginate/powder ratio of 1:15 (6.7 wt% of alginate). The mixture was extruded dropwise at room temperature into a 0.15 M CaCl_2_ solution, using a 0.70 mm diameter needle. The obtained microspheres were mature in the CaCl_2_ solution for 24 h for complete agglutination. The nCHA microspheres were dried overnight in an oven at 30 °C and separated using sieves with the desired mesh.

### 2.5. Preparation of Biomaterial for Grafting

The rhBMP-2 was subjected to the preparation according to the manufacturer’s guidelines. After appropriate reconstitution, the concentration of rhBMP-2 is 1.5 mg/mL. The solution is then applied to the provided absorbable collagen sponge. Infuse™ Bone Graft was prepared at the time of surgery and allowed a prescribed amount of time (no less than 15 min) before placement at the rat calvaria. Half of the solution was used with half absorbable collagen sponge and the other half solution was mixed to the microspheres (150 mg) and then grafted in five rats calvarias. The COL/BMP-2 group was composed of a 7.5 mL of rhBMP-2 solution associated with the half collagen membrane contained in the kit. The nCHA/BMP-2 group was composed of a standardized mass of nCHA (150 mg) and 7.5 mL of BMP-2 for the animal’s implantation.

### 2.6. Anesthetic and Surgical Procedures

For the experimental surgical procedures, the animals were deprived of the diet six hours before the surgery and submitted to general anesthesia, receiving 100 mg/kg of ketamine IM (Virbac^®^, Veltbrands, São Paulo, Brazil), 10 mg/kg of xylazine (Sedazine^®^, Fort Dodge, Rio de Janeiro, Brazil), and 5 mg/kg of Midazolam (Eurofarma, Rio de Janeiro, Brazil) intraperitoneally. Each animal received a 3mL dose of a solution.

After observing the absence of pain reflexes, degermation, trichotomy, and antisepsis of calvaria were conducted. The animals were taken to an operating table of their own, and the sterilized surgical fields were positioned. A semilunar incision was made on each animal’s calvaria using a 15C knife scalpel (Becton-Dickinson^®^, Curitiba, Brazil). After the incision, the subperiosteal detachment was followed with exposure of the desired bone area. A critical-sized surgical defect was performed with an 8 mm diameter trephine bur (SIN^®^, São Paulo, Brazil) engaged in an implant handpiece under copious irrigation with sterile saline. The defects were filled with respective treatments: only blood (clot), collagen membrane, that was cut with the trephine bur so that its size was compatible with the defect, and BMP2 (COL/rhBMP-2) and microspheres of nanostructured carbonated hydroxyapatite (nCHA/rhBMP-2). The defects were filled only to the surrounding bone level without packing and gently placed in the defects without displacing the dura. Finally, the skin was sutured in a single plane with 5.0 Nylon (Technofio, Permed, Mafra, Santa Catarina, Brazil), and the surgical wound remained uncovered. A surgeon, with experience in calvaria surgeries, conducted all surgical procedures.

To control pain, the animals received anti-inflammatory by intramuscular injection of Maxicam^®^ (Maxicam^®^, Ourofino pet—Osasco, São Paulo, Brazil) 1 mL/kg, starting on the first day and continuing for another two days. After this procedure, the animals were returned to the mini isolators, receiving feed and water at will for recovery. After 45 days post-surgery, the animals were euthanized with a lethal dose of general anesthetic to collect the samples.

### 2.7. Histological Process

The bone blocks were containing the defects were dissected, removed with a margin of safety of approximately 5 mm on each side of the implantation region. After, the samples were fixed in a 4% formaldehyde phosphate-buffered at pH 7.2 for 48 h. Calvaria samples’ decalcification occurred with 10% buffered ethylenediaminetetraacetic acid (EDTA) for two days at room temperature. Finally, the samples were embedded in paraffin and cut to obtain sections of 5 µm, stained with Hematoxylin and Eosin (HE). One sample of each group was not decalcified but embedded in resin (Technovit 7200 VLC, Kultzer & Co., Wehrheim, Germany). A region in the middle of the defect was selected for cutting and processing until obtaining a cylindrical shape with a diameter of approximately 1.5 mm for SR-µCT analyzes.

### 2.8. Image Acquisition by SR-µCT and 3D Segmentation and Processing

The images were acquired in the IMX Beamline at Brazilian Synchrotron Light Laboratory (LNLS). The source is a quasi-parallel polychromatic X-ray beam with a peak at 15 keV and bandwidth of 7.7 keV, after being filtered by a 350 µm Si filter. A total of 1024 projections in a 180° turn were collected using an optical system composed by a 50 µm LuAG:Ce Scintillator, a 10× objective lens and a 14-bit CCD camera PCO.2000. The equivalent pixel size was 0.82 × 0.82 µm^2^ with a field of view of 1.68 × 1.68 mm^2^. A fast image reconstruction algorithm, Raft [26], was used to reconstruct the set of the 2D projections into a 3D image with a voxel size of 0.82 × 0.82 × 0.82 µm^3^. A total of six tomograms at different heights of the sample was required to cover the entire region of interest (all the defect). The set of tomograms was filtered, merged, segmented, and processing using Avizo 9.7 software (Huston, TX, USA). The images were segmented using mainly the interactive watershed method, classifying both the new and pre-existing bone phase.

### 2.9. Histological Analysis

The slides obtained from the decalcified blocks and HE-stained were observed under an optical microscope (OLYMPUS BX43, Tokyo, Japan). The images were captured using a high-resolution digital camera (OLYMPUS SC100, Tokyo, Japan). The descriptive analysis of the closing of the bone defect, presence of osteoid matrix, inflammatory infiltrates, reminiscent biomaterial, and progression of the type of healing presented in bone defect was evaluated.

### 2.10. Histomorphometric Evaluation

The histological calvaria slices were examined under a light microscope. Six non-superimposing photomicrographs were captured at 40× magnification from each slide, corresponding to the interest regions covering both defects edges. The histomorphometry was performed using Image-Pro Plus^®^ 6.0 software (Media Cybernetics, Silver Spring, MD, USA), which generates a grid of 250 points that allowed the newly formed bone and connective tissue volume densities to be determined [9,27]. The obtained values were transferred to a database developed using Microsoft Excel^®^ spreadsheet software (Seattle, WA, USA) for subsequent statistical analysis.

### 2.11. Statistical Analysis

The data were expressed as means and confidence intervals. A quantitative description of newly formed bone and connective tissue volume density (%) was done by parametric description with means and confidence intervals (CI). After applying the Shapiro–Wilk normality test, the data were transformed into a Y logarithm, and the variability of the measures was evaluated with a significance level of *p* < 0.05. The analysis of variance (ANOVA) and Tukey’s post-test was applied to investigate the statistical differences between treatments. The Prism Graph Pad 8.0 software (La Jolla, CA, USA) allowed the statistical analysis.

## 3. Results

### 3.1. Descriptive Histological Analysis

**Figure 1:**
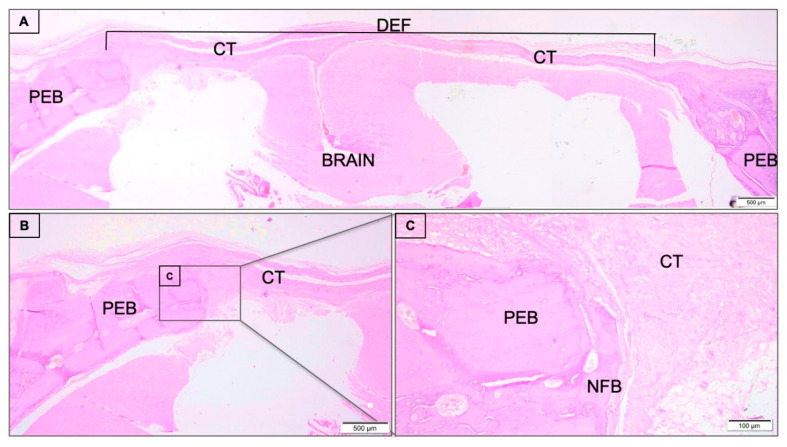
(**A**) Histological sections of critical size calvaria defect of clot group 45 days post-surgery. The calvaria defect (DEF) occupied by connective tissue (CT), inflammatory infiltrate and a scarce band of newly formed bone (NFB) in the periphery of the defect. The small square positioned in defect are displayed at 20-fold magnification (**B**) adjacent to the figure with lower magnification (4-fold magnification) (**C**). Preexisting bone (PEB). Histological section stained with hematoxylin/eosin.

The clot group presented the area of the critical defect site filled exclusively by connective tissue composed of collagen fibers, absence of newly formed bone at the center of the defect, and minimal presence of bone formation at the margin of the defect (Figure 1).

**Figure 2:**
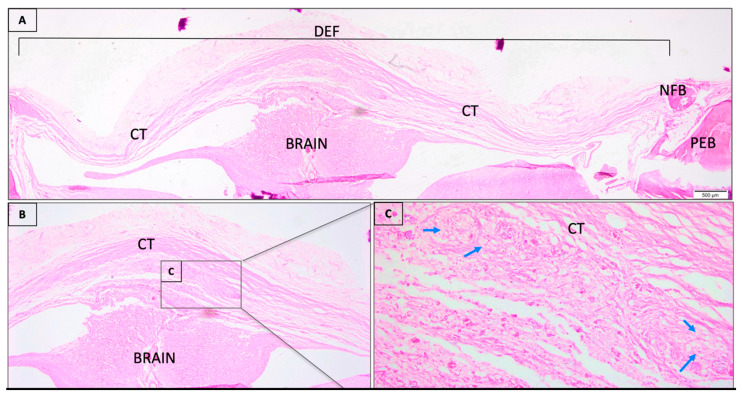
(**A**) Histological sections of critical size calvaria defect of COL/rhBMP-2 group 45 days post-surgery. The calvaria defect (DEF) occupied by connective tissue (CT), inflammatory infiltrate predominated by macrophages (blue arrows) and a scarce band of newly formed bone (NFB) at the periphery of the defect. The small square positioned in defect are displayed at 20-fold magnification (**B**) adjacent to the figure with lower magnification (4-fold magnification) (**C**). Preexisting bone (PEB). Histological section stained with hematoxylin/eosin.

The critical bone defects in calvaria filled with COL/BMP2 presented the interest area filled by connective tissue band (Figure 2A) and scarce new-formed bone present in the periphery of the defect involved by small inflammatory cells (Figure 2B,C and Figure S1).

**Figure 3:**
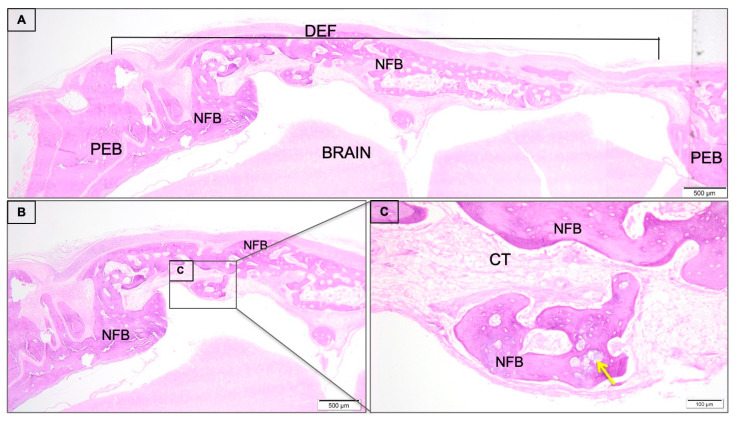
(**A**) Histological sections of critical size calvaria defect of nCHA/rhBMP-2 group 45 days post-surgery. The calvaria defect (DEF) newly formed bone (NFB) with trabecular aspect permeated by connective tissue (CT). Presence of remaining biomaterial (yellow arrow). Peripherally to the defect presence of preexisting bone (PEB). The small square positioned in defect are displayed at 20-fold magnification. (**B**) adjacent to the figure with lower magnification (4-fold magnification) (**C**). Histological section stained with hematoxylin/eosin.

The nCHA/BMP-2 occupied the region of critical defect predominantly filled by new-formed bone trabeculae, almost filling the bone bridge of the defect (Figure 3A). The bone trabeculae were interspersed by connective tissue. Additionally, it evidenced the presence of residual biomaterial into the bone trabeculae (Figure 3B,C).

### 3.2. SRµCT Results

**Figure 4:**
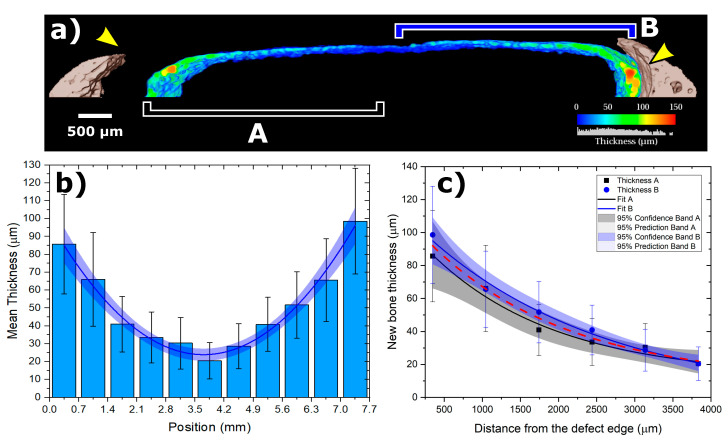
CHA/rhBMP-2 treated calvaria defect after 45 days. (**a**) Volume Rendering of calvaria (coronal cross-section view), observe the presence of new bone band over most of the defect; yellow arrows represent the extremes of the defect; the colored thickness map indicates the local new-bone thickness along with the defect. (**b**) The newly formed bone thickness behavior according to the bone defect sites (A, black; B, blue). (**c**) New bone thickness vs. the distance from the defect edge, fitted by an exponential decaying with 95% prediction and confidence bands. The black color represents region A, and the blue, region B, as indicated in (**a**). Micrometer bar: 500 μm.

Through the qualitative analysis of SR-μCT, in the nCHA/BMP-2 group, it was possible to note the presence of a newly formed bone band in the extent of the critical defect (Figure 4a). Figure 4b shows the entire defect together with the new bone phase as a thickness mapping of the newly formed bone throughout the entire defect region. The behavior of the thickness of the newly formed bone describes an exponential decay as the distance to the edge increases, i.e., growing towards the center of the defect (Figure 4c). The thickness as a function of distance is given by:

$$Th\left(d\right)\;=\;a+A\;e^{-d/t},$$

where a = 12.8±7.3 µm; A = 99.2±3.7 µm; *e* t = 1817±236.5, as shown in Figure 4c.

**Figure 5:**
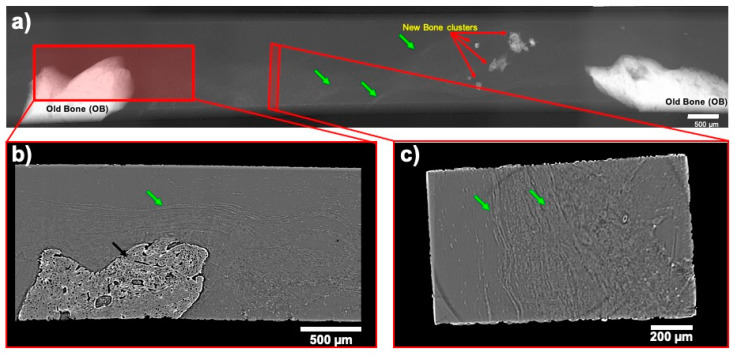
Coronal cross-section of Membrane + rhBMP-2 group. (**a**) Corrected projection of the defect gap; (**b**) In-plane SR-µCT reconstructed slice. (**c**) perpendicular plane SR-µCT showing the connective tissue. Black arrow: bone; green arrows: connective tissue; red arrows: new bone formation clusters.

The analysis of SR-μCT, in the COL/rhBMP-2 group, it was possible to note the absence of a newly formed bone in the extent of the critical defect (Figure 5).

### 3.3. Histomorphometric Results

**Figure 6:**
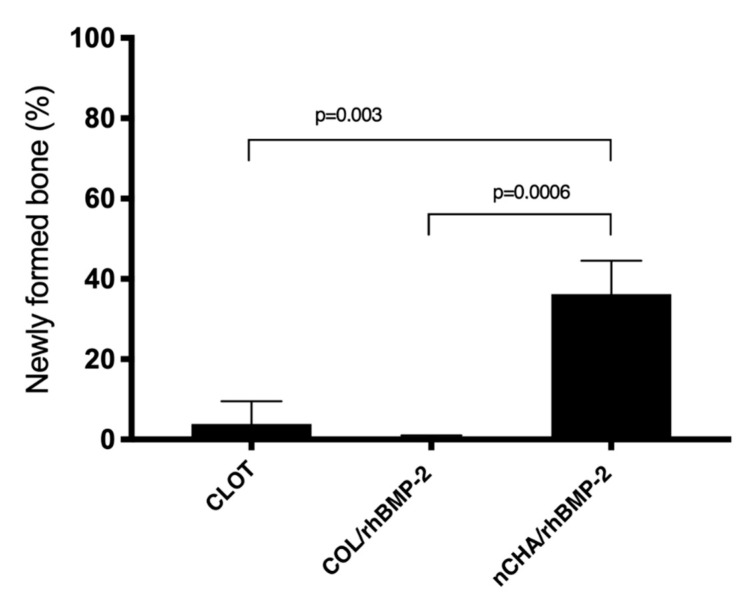
New formed bone volume density (%) of CLOT, COL/rhBMP-2 and nCHA/rhBMP-2 groups 45 days after surgery. The horizontal bar represents significant statistical difference between different treatments (ANOVA and Tukey’s post-test, *p* < 0.05). Results are shown as mean percentage ± confidence interval. The results are representative of 5 mice/group.

Figure 6 presents a means and confidence interval of newly formed bone volume density (%) of CLOT, COL/rhBPM-2, and nCHA/rhBMP-2 groups after 45 days. nCHA/rhBMP-2 group (36.24 ± 6.68) showed a significant increase in newly formed bone (almost 40% of the defect) compared to COL/BPM-2 (0.42 ± 0.40) (*p* = 0.0006). Additionally, the nCHA/rhBMP-2 presented almost 10-fold higher new-formed bone than the clot group clot (3.84 ± 4.57) (*p* = 0.003) (*p* < 0.05).

**Figure 7:**
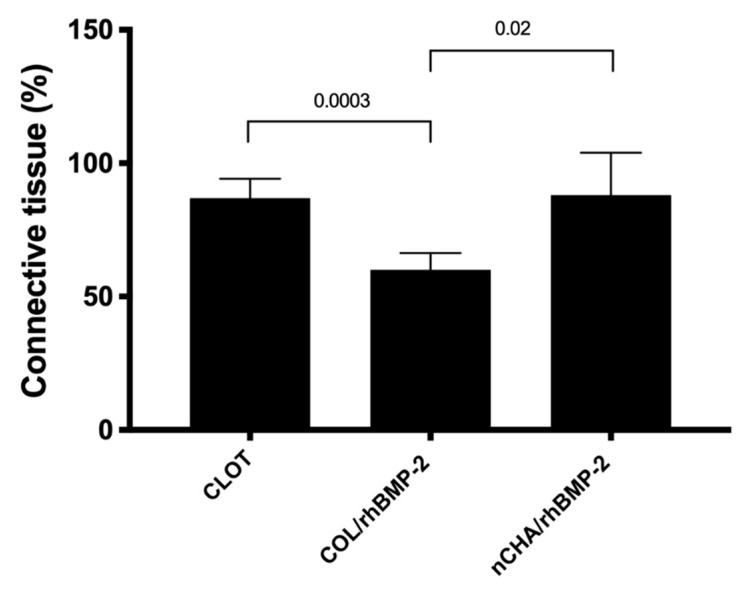
Connective tissue volume density (%) of CLOT, COL/rhBMP-2 and nCHA/rhBMP-2 groups 45 days after surgery. The horizontal bar represents significant statistical difference between different treatments (ANOVA and Tukey’s post-test, *p* < 0.05). Results are shown as mean percentage ± confidence interval. The results are representative of 5 mice/group.

The connective tissue volume density (%) of CLOT, COL/rhBPM-2, and nCHA/rhBMP-2 groups are presented in Figure 7. After 45 days, the CLOT group (86.96 ± 5.83) and nCHA/rhBMP-2 (88.04 ± 12.83) presented a significant amount of connective tissue compared to COL/rhBPM-2 (60.10 ± 4.95).

## 4. Discussion

Animals models have been widely used to investigate the use of biomaterials for bone regeneration. The choice of animal models frequently takes the phylogenetic tree into account, although, if trustful data can be achieved using small animals, such as rodents, it is desirable [28]. Concerning bone regeneration, rodents can have many advantages, such as a better cost–benefit ratio and ease to house and handle. Biomaterials can be inserted with adequate surgical access without the need for external fixation owing to the support provided by the dura mater and the skin, evoke little social concern, and enable the normalization of experimental conditions in genetically similar individuals [29,30]. In rats, calvaria’s critical size defect is one of the most commonly used experimental models for assessing bone healing. It was first described as “the smallest size intra-osseous wound in a particular bone and species that will not heal spontaneously during the life-time of the animal” [31]. A recent systematic review concluded that a defect in the calvaria with a 5 mm diameter or more could be considered a critical size defect [32]. In this study, we used an 8 mm defect that is considered a critical size defect for rats calvaria, allowing the direct comparison to other studies [10,32,33].

Several biomaterials have been considered and used as bone substitutes, mostly synthetic calcium phosphate ceramics are revolutionizing research and treatments on bone tissue engineering [2]. Among calcium phosphates, HA can be distinguished from other calcium phosphates by its similarity to the inorganic part of human bone, unlimited amount, biocompatibility, and suitable osteoconductive property. However, its uses are limited by its high crystallinity and low absorption rate, a previously observed limitation when HA is synthesized and subjected to a high-temperature treatment [4]. Radical substitution, particle size, porosity, and shape can also interfere in the inflammatory response and the new bone formation [34].

Our study evaluated the nanostructured carbonated hydroxyapatite (nCHA), which was presented in previous pre-clinical and clinical studies, biocompatibility, and better rates of bioabsorption, characteristics that contribute to bone repair [7,10,35]. The microspheres were synthesized at 37 °C and were not sintered, and its physicochemical characterization was previously [11,35] evaluated with scanning electron microscopy, that showed its morphological characteristics, the Fourier transform infrared (FTIR) spectroscopy, which presented intense water bands, phosphate ions, and carbonate ions, confirming that the replacements occurred as expected, and the X-ray diffractogram showed peaks corresponding to standard hydroxyapatite. Habibovic et al. [5] also observed these results, describing that carbonated HA produces more favorable biological responses because carbonates in the apatite network structure increase the chemical reactivity, facilitating the resorption of bone tissue. This biomaterial strongly resembles natural apatite, improving its performance [5,7]

Regarding the spere shape used in the presented study, this microstructure is reported in the literature suggesting a lower inflammatory response than the needle format [13]. The spheres used have a diameter from 425 to 600 µm, which in previous studies concluded that granules of 250–500 µm in size might be a more suitable scaffold [36]. Additional characterizations of nCHA were reported before [10,37], which presented through synchrotron radiation-based X-ray microtomography (SR-μCT) microspheres made up of agglomerates of nanoparticles, and the interconnected pore spaces were filled with the sodium alginate polymer. The porous volume of nCHA was 23.8%, with average equivalent pore diameters of 5.54 µm. This porous characteristic enhances the biomaterial surface’s specific surface area, which is a decisive parameter for the dissolution rates favoring the cellular population [10].

The nCHA has excellent osteoconductive properties; meanwhile, it has not osteoinductive capacity, limiting its application to repair large bone defects or critical-sized bone defects [38]. Meaningful progress in treating bone defects has been the introduction of rhBMPs, specifically rhBMP-2, because of its osteoinduction properties. The rhBMP-2 release creates an osteogenic microenvironment that allows multipotent cell progenitors to migrate to the area of injury and proliferate and differentiate toward the osteogenic lineage [17,18]. However, carriers enhancing the binding of BMPs are of the most importance. The four major categories of rhBMPs carrier materials are natural polymers, inorganic materials, synthetic polymers, and these materials’ composites. This study used a composite biomaterial with a porous nCHA microsphere technology as a carrier for rhBMP-2. This could meet the need for bone graft substitutes that combined osteoconductive properties of calcium phosphates to bone morphogenetic proteins [39].

A previous study [39] investigated the ability of hollow hydroxyapatite (HA) microspheres to serve as a carrier for the controlled release of rhBMP-2 in a 4.6 mm in diameter calvaria rat defect. They conclude that, with a high surface area, rhBMP-2-loaded microspheres presented a significantly better capacity to regenerate bone at 3 and 6 weeks than those without rhBMP-2, 40%, and 43%, compared to 13% and 17%, respectively. Our histomorphometric results of nCHA showed similar values of NFB, however, it is essential to consider that, in our study, we used a critical size defect model, which showed a bone regeneration of 36.24 ± 6.68 when compared to the group of collagen membranes with rhBMP-2 (0.42 ± 0.40).

In a previous study in rat calvaria, the authors evaluated the newly formed bone after 1, 3, and 6 months for nCHA without rhBMP-2 [10]. The histomorphometric analysis showed that the newly formed bone volume was limited to regions close to the preexistent bone, whereas connective tissue occupied the central part of the defect. These results stimulated the interest in associating nCHA to an osteoinductive material as rhBMP-2.

According to Choi et al. [40], in a preclinical study on calvaria defects in the beagle dog, which evaluated the dosage based on the extent defect treated with BMP-2 and β-tricalcium phosphate scaffolds after 16 weeks, the proportion of biomaterial/rhBMP-2 may determine the local hyperosteogenesis or hypo-osteogenesis results. From these results a new study evaluating different dosages of rhBMP-2 for rat calvaria should be conducted to clarify the best biomaterial-rhBMP-2 ratio in this model.

According to Issa et al. [19], it was observed that the association of the graft to rhBMP-2 was able to increase bone formation due to the synergism between the graft and the protein that is involved in osteoconduction, osteoinduction, and osteopromotion. It is also believed that the graft may locally maintain active protein for longer, serving as a carrier for delivering the protein to the medium slowly, accelerating bone repair.

## 5. Conclusions

The results demonstrated that the association of nCHA/rhBMP-2 optimizes bone repair compared to the other groups evaluated in this study, evidencing the efficiency of nCHA as a carrier of rhBMP-2 and presenting superior results to the collagen membrane, a material currently used as a vehicle for the clinical application of rhBMP-2.

## Supplementary Materials

The following are available online at https://www.mdpi.com/2079-4983/11/4/87/s1, Figure S1. Photomicrograph representative of inflammatory infiltrate predominated by macrophages (blue arrows) of COL/rhBMP-2 group. Magnification: 40×; Stain: Hematoxylin and Eosin.

## References

[1] De Fernandez Grado G., Keller L., Idoux-Gillet Y., Wagner Q., Musset A.M., Benkirane-Jessel N., Bornert F., Offner D. (2018). Bone substitutes: A review of their characteristics, clinical use, and perspectives for large bone defects management. J. Tissue Eng..

[2] Sadat-Shojai M., Khorasani M.T., Dinpanah-Khoshdargi E., Jamshidi A. (2013). Synthesis methods for nanosized hydroxyapatite with diverse structures. Acta Biomater..

[3] Ratnayake J.T.B., Mucalo M., Dias G.J. (2017). Substituted hydroxyapatites for bone regeneration: A review of current trends. J. Biomed. Mater. Res. B Appl. Biomater..

[4] Resende R.F., Fernandes G.V., Santos S.R., Rossi A.M., Lima I., Granjeiro J.M., Calasans-Maia M.D. (2013). Long-term biocompatibility evaluation of 0.5% zinc containing hydroxyapatite in rabbits. J. Mater. Sci. Mater. Med..

[5] Habibovic P., Juhl M.V., Clyens S., Martinetti R., Dolcini L., Theilgaard N., van Blitterswijk C.A. (2010). Comparison of two carbonated apatite ceramics in vivo. Acta Biomater..

[6] Valiense H., Barreto M., Resende R.F., Alves A.T., Rossi A.M., Mavropoulos E., Granjeiro J.M., Calasans-Maia M.D. (2016). In vitro and in vivo evaluation of strontium-containing nanostructured carbonated hydroxyapatite/sodium alginate for sinus lift in rabbits. J. Biomed. Mater. Res. B Appl. Biomater..

[7] Calasans-Maia M.D., Melo B.R., Alves A.T., Resende R.F., Louro R.S., Sartoretto S.C., Granjeiro J.M., Alves G.G. (2015). Cytocompatibility and biocompatibility of nanostructured carbonated hydroxyapatite spheres for bone repair. J. Appl. Oral Sci..

[8] Carmo A.B.X.D., Sartoretto S.C., Alves A.T.N.N., Granjeiro J.M., Miguel F.B., Calasans-Maia J., Calasans-Maia M.D. (2018). Alveolar bone repair with strontium-containing nanostructured carbonated hydroxyapatite. J. Appl. Oral Sci..

[9] Sartoretto S.C., Calasans-Maia M.D., Alves A.T.N.N., Resende R.F.B., da Costa Fernandes C.J., de Magalhães Padilha P., Rossi A.M., Teti A., Granjeiro J.M., Zambuzzi W.F. (2020). The role of apoptosis associated speck-like protein containing a caspase-1 recruitment domain (ASC) in response to bone substitutes. Mater. Sci. Eng. C Mater. Biol. Appl..

[10] Martinez-Zelaya V.R., Zarranz L., Herrera E.Z., Alves A.T., Uzeda M.J., Mavropoulos E., Rossi A.L., Mello A., Granjeiro J.M., Calasans-Maia M.D. (2019). In Vitro and in vivo evaluations of nanocrystalline Zn-doped carbonated hydroxyapatite/alginate microspheres: Zinc and calcium bioavailability and bone regeneration. Int. J. Nanomed..

[11] de Almeida Barros Mourão C.F., Lourenço E.S., Nascimento J.R.B., Machado R.C.M., Rossi A.M., Leite P.E.C., Granjeiro J.M., Alves G.G., Calasans-Maia M.D. (2019). Does the association of blood-derived growth factors to nanostructured carbonated hydroxyapatite contributes to the maxillary sinus floor elevation? A randomized clinical trial. Clin. Oral Investig..

[12] Calasans-Maia M.D., Barboza Junior C.A.B., Soriano-Souza C.A., Alves A.T.N.N., Uzeda M.J.P., Martinez-Zelaya V.R., Mavropoulos E., Rocha Leão M.H., de Santana R.B., Granjeiro J.M. (2019). Microspheres of alginate encapsulated minocycline-loaded nanocrystalline carbonated hydroxyapatite: Therapeutic potential and effects on bone regeneration. Int. J. Nanomed..

[13] Lebre F., Sridharan R., Sawkins M.J., Kelly D.J., O’Brien F.J., Lavelle E.C. (2017). The shape and size of hydroxyapatite particles dictate inflammatory responses following implantation. Sci. Rep..

[14] Chen G., Deng C., Li Y.P. (2012). TGF-β and BMP signaling in osteoblast differentiation and bone formation. Int. J. Biol. Sci..

[15] Gautschi O.P., Frey S.P., Zellweger R. (2007). Bone morphogenetic proteins in clinical applications. ANZ J. Surg..

[16] Granjeiro J.M., Oliveira R.C., Bustos-Valenzuela J.C., Sogayar M.C., Taga R. (2005). Bone morphogenetic proteins: From structure to clinical use. Braz. J. Med. Biol. Res..

[17] Sheikh Z., Javaid M.A., Hamdan N., Hashmi R. (2015). Bone Regeneration Using Bone Morphogenetic Proteins and Various Biomaterial Carriers. Materials (Basel).

[18] Bessa P.C., Casal M., Reis R.L. (2008). Bone morphogenetic proteins in tissue engineering: The road from laboratory to clinic, part II (BMP delivery). J. Tissue Eng. Regen. Med..

[19] Issa J.P., Bentley M.V., Iyomasa M.M., Sebald W., de Albuquerque R.F. (2008). Sustained release carriers used to delivery bone morphogenetic proteins in the bone healing process. Anat. Histol. Embryol..

[20] David L., Feige J.J., Bailly S. (2009). Emerging role of bone morphogenetic proteins in angiogenesis. Cytokine Growth Factor Rev..

[21] Carreira A.C., Lojudice F.H., Halcsik E., Navarro R.D., Sogayar M.C., Granjeiro J.M. (2014). Bone morphogenetic proteins: Facts, challenges, and future perspectives. J. Dent. Res..

[22] Kilkenny C., Browne W., Cuthill I.C., Emerson M., Altman D.G. (2010). NC3Rs Reporting Guidelines Working Group. Animal research: Reporting in vivo experiments: The ARRIVE guidelines. Br. J. Pharmacol..

[23] Smith A.J., Clutton R.E., Lilley E., Hansen K.E.A., Brattelid T. (2018). PREPARE: Guidelines for planning animal research and testing. Lab. Anim..

[24] Luvizuto E.R., Tangl S., Zanoni G., Okamoto T., Sonoda C.K., Gruber R., Okamoto R. (2011). The effect of BMP-2 on the osteoconductive properties of β-tricalcium phosphate in rat calvaria defects. Biomaterials.

[25] Charan J., Kantharia N.D. (2013). How to calculate sample size in animal studies?. J. Pharmacol. Pharmacother..

[26] Miqueles G., Martinez G., Guerrero Prado P. Fast image reconstruction at a synchrotron laboratory. Proceedings of the 2020 SIAM Conference on Parallel Processing for Scientific Computing.

[27] Sartoretto S., Gemini-Piperni S., da Silva R.A., Calasans M.D., Rucci N., Pires Dos Santos T.M., Lima I.B.C., Rossi A.M., Alves G., Granjeiro J.M. (2019). Apoptosis-associated speck-like protein containing a caspase-1 recruitment domain (ASC) contributes to osteoblast differentiation and osteogenesis. J. Cell. Physiol..

[28] Gomes P.S., Fernandes M.H. (2011). Rodent models in bone-related research: The relevance of calvarial defects in the assessment of bone regeneration strategies. Lab. Anim..

[29] Jordan H.V. (1971). Rodent model systems in peri-odontal disease research. J. Dent. Res..

[30] Pellegrini G., Seol Y.J., Gruber R., Giannobile W.V. (2009). Preclinical models for oral and peri-odontal reconstructive therapies. J. Dent. Res..

[31] Schmitz J.P., Hollinger J.O. (1986). The critical size defect as an experimental model for cranio-mandibulofacial nonunions. Clin. Orthop. Relat. Res..

[32] Vajgel A., Mardas N., Farias B.C., Petrie A., Cimoes R., Donos N. (2014). A systematic review on the critical size defect model. Clin. Oral Implants Res..

[33] Cuozzo R.C., Sartoretto S.C., Resende R.F.B., Alves A.T.N.N., Mavropoulos E., da Prado Silva M.H., Calasans-Maia M.D. (2020). Biological evaluation of zinc-containing calcium alginate-hydroxyapatite composite microspheres for bone regeneration. J. Biomed. Mater. Res. B Appl. Biomater..

[34] de MateSanchez Val J.E., Calvo-Guirado J.L., Gomez-Moreno G., Martınez C.P.-A., Mazon P., De Aza P.N. (2016). Influence of hydroxyapatite granule size, porosity, and crystallinity on tissue reaction in vivo. Part A: Synthesis, characterization of the materials, and SEM analysis. Clin. Oral Implants Res..

[35] Resende R.F.B., Sartoretto S.C., Uzeda M.J., Alves A.T.N.N., Calasans-Maia J.A., Rossi A.M., Granjeiro J.M., Calasans-Maia M.D. (2019). Randomized Controlled Clinical Trial of Nanostructured Carbonated Hydroxyapatite for Alveolar Bone Repair. Materials (Basel).

[36] Coathup M.J., Cai Q., Campion C., Buckland T., Blunn G.W. (2013). The effect of particle size on the osteointegration of injectable silicate-substituted calcium phosphate bone substitute materials. J. Biomed. Mater. Res. Part B.

[37] Martinez-Zelaya V.R., Archilha N.L., Calasans-Maia M., Farina M., Rossi A.M. (2020). Trabecular architecture during the healing process of a tibial diaphysis defect. Acta Biomater..

[38] Cancedda R., Giannoni P., Mastrogiacomo M. (2007). A tissue engineering approach to bone repair in large animal models and in clinical practice. Biomaterials.

[39] Xiao W., Fu H., Rahaman M.N., Liu Y., Bal B.S. (2013). Hollow hydroxyapatite microspheres: A novel bioactive and osteoconductive carrier for controlled release of bone morphogenetic protein-2 in bone regeneration. Acta Biomater..

[40] Choi J.W., Jeong W.S., Yang S.J., Park E.J., Oh T.S., Koh K.S. (2016). Appropriate and Effective Dosage of BMP-2 for the Ideal Regeneration of Calvarial Bone Defects in Beagles. Plast. Reconstr. Surg..

