# Supplementary material for: Nanostructured Carbonated Hydroxyapatite Associated to rhBMP-2 Improves Bone Repair in Rat Calvaria
Source: J Funct Biomater. 2020 Dec 4;11(4):87. doi: 10.3390/jfb11040087 (PMC7768361; doi:10.3390/jfb11040087)
Supplement: Supplementary file 1 [file jfb-11-00087-s001.pdf]

Journal of
Functional
Biomaterials

Supplementary File

Nanostructured Carbonated Hydroxyapatite Associated to rhBMP-2 Improves Bone Repair in Rat Calvaria

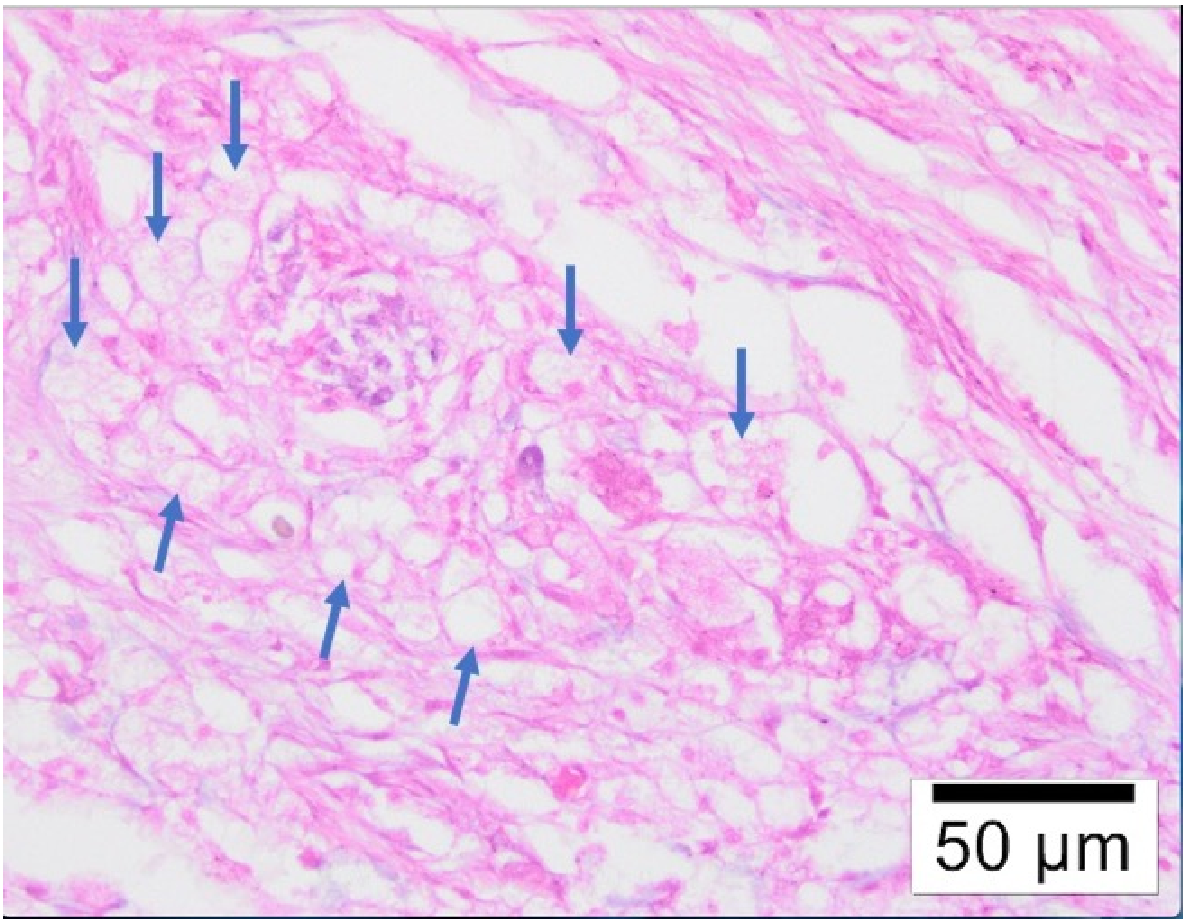

Figure S1. Photomicrograph representative of inflammatory infiltrate predominated by macrophages (blue arrows) of COL/rhBMP-2 group. Magnification: 40×; Stain: Hematoxylin and Eosin.
